# Human genes with relative synonymous codon usage analogous to that of polyomaviruses are involved in the mechanism of polyomavirus nephropathy

**DOI:** 10.3389/fcimb.2022.992201

**Published:** 2022-09-08

**Authors:** Yu Fan, Duan Guo, Shangping Zhao, Qiang Wei, Yi Li, Tao Lin

**Affiliations:** ^1^ Department of Urology, National Clinical Research Center for Geriatrics and Organ Transplantation Center, West China Hospital of Sichuan University, Chengdu, China; ^2^ Department of Palliative Medicine, West China School of Public Health and West China fourth Hospital, Sichuan University, Chengdu, China; ^3^ Palliative Medicine Research Center, West China−Peking Union Medical College, Chen Zhiqian (PUMC C.C). Chen Institute of Health, Sichuan University, Chengdu, China; ^4^ Department of Urology, West China School of Nursing and Organ Transplantation Center, West China Hospital of Sichuan University, Chengdu, China; ^5^ Department of Laboratory Medicine, West China Hospital, Sichuan University, Chengdu, China

**Keywords:** human polyomavirus, relative synonymous codon usage, codon usage bias, polyomavirus nephropathy, molecular mechanism

## Abstract

Human polyomaviruses (HPyVs) can cause serious and deleterious infections in human. Yet, the molecular mechanism underlying these infections, particularly in polyomavirus nephropathy (PVAN), is not well-defined. In the present study, we aimed to identify human genes with codon usage bias (CUB) similar to that of HPyV genes and explore their potential involvement in the pathogenesis of PVAN. The relative synonymous codon usage (RSCU) values of genes of HPyVs and those of human genes were computed and used for Pearson correlation analysis. The involvement of the identified correlation genes in PVAN was analyzed by validating their differential expression in publicly available transcriptomics data. Functional enrichment was performed to uncover the role of sets of genes. The RSCU analysis indicated that the A- and T-ending codons are preferentially used in HPyV genes. In total, 5400 human genes were correlated to the HPyV genes. The protein-protein interaction (PPI) network indicated strong interactions between these proteins. Gene expression analysis indicated that 229 of these genes were consistently and differentially expressed between normal kidney tissues and kidney tissues from PVAN patients. Functional enrichment analysis indicated that these genes were involved in biological processes related to transcription and in pathways related to protein ubiquitination pathway, apoptosis, cellular response to stress, inflammation and immune system. The identified genes may serve as diagnostic biomarkers and potential therapeutic targets for HPyV associated diseases, especially PVAN.

## Introduction

Human polyomaviruses (HPyVs) are a group of 13 viruses characterized by a circular double-stranded DNA infecting a significant margin (30%-90%) of the human population worldwide (Dalianis and Hirsch, 2013; Wu et al., 2021). HPyVs are endowed with a characteristic genetic material allowing them to produce viral proteins such as the large and small T antigens in the early region and the proteins of the capsid (VP1, 2, and 3) (Moens et al., 2007; Burkert et al., 2014; Cook, 2016). Among the HPyVs, 11 are known to frequently infect humans, and six of them are linked with human diseases (Ahsan and Shah, 2006; Klufah et al., 2021). Nevertheless, little is known about the molecular mechanisms involved in the pathogenesis of infections due to these viruses.

Studies have indicated that, following infection, HPyVs can integrate in the genome and persist in various tissues such as spleen, brain and kidneys (Feng et al., 2008; Bertz et al., 2020; Wang et al., 2020; Jin et al., 2021); these viruses can be reactivated from latency and infect additional tissues in immunocompromised patients (Wiedinger et al., 2014; Yang and You, 2020). The most documented HPyVs are BKPyV (HPyV1 or *Betapolyomavirus hominis*) associated with PVAN (PVAN) in renal transplant recipients, late-onset hemorrhagic cystitis, ureteral stenosis, and bladder cancer (Hirsch and Randhawa, 2019; Blackard et al., 2020; Furmaga et al., 2021), the JCPyV (also known as HPyV2) that causes progressive multifocal leukoencephalopathy and JCPyV-associated nephropathy (L'Honneur and Rozenberg, 2016; Assetta and Atwood, 2017; Morris-Love et al., 2019; Harypursat et al., 2020; Multani and Ho, 2020), and the MCPyV (also known as HPyV5) involved in Merkel cell carcinoma (MCC) (Becker et al., 2009; Babakir-Mina et al., 2011; Becker et al., 2017; Becker et al., 2019; Pietropaolo et al., 2020). The other HPyVs that can cause infections in human include HPyV4 (WUPyV), HPyV9, HPyV3 (KIPyV) and Trichodysplasia spinulosa PyV (TSPyV) (Babakir-Mina et al., 2013; Nicol et al., 2013; Rao et al., 2016; van der Meijden and Feltkamp, 2018).

The infection of kidney tissues by HPyVs is associated with complications such as PVAN and allograft rejection. BKPyV can simultaneously induce inflammatory and fibrotic responses, immunosuppression, and tubular injury in kidney transplants, thereby causing kidney transplant rejection in 40–70% of infected grafts (Babel et al., 2011; Ambalathingal et al., 2017; Raupp et al., 2020; Kotla et al., 2021; Shen et al., 2021). JCPyV-associated PVAN occurs in <3% of PVAN cases after renal transplantation (Kantarci et al., 2011; Yang et al., 2017; Höcker et al., 2018). MCPyV, HPyV9 and TSPyV viremia has been detected in BKPyV infection and BKPyV-induced PVAN renal allograft patients, but their impact on the infectious pathogenesis is not elucidated (van Rijn et al., 2019; Wang et al., 2019b). Sustained TSPyV viremia indicating its infection of kidney transplant recipient has been also reported (Zanella et al., 2021). JCPyV, TSPyV, MCPyV and HPyV9 have been detected in allograft kidneys and in urine after transplantation (van der Meijden et al., 2014; Kamminga et al., 2021). However, the mechanism of HPyVs in HPyV-induced nephropathy and in transplant rejection remains unknown. Moreover, the pathogenesis of PVAN remains to be elucidated. Thus, clarifying the molecular mechanism of HPyV infections could benefit, not only the diagnosis and monitoring of patients with PVAN, but also other infectious diseases related to human HPyVs.

Studies indicate that viral infection impact profoundly on the gene expression system in host cells which mobilize particular gene expression pathways as a defense response to viral infection (Tripp et al., 2013; Yu et al., 2019; Das et al., 2021; Tsalik et al., 2021). Up to date, the identification of genes involved in host response to viral infections is based majorly on the transcriptomics and proteomics studies (Flentje et al., 2018; Wang et al., 2019a; Yao et al., 2019; Ahmed, 2020; Xiong et al., 2020; Maróti et al., 2021). Interestingly, studies indicate that the co-evolution of the viruses and their hosts can affect gene expression in the hosts (Nambou and Anakpa, 2020; Das et al., 2021; Nambou et al., 2022). However, no study has explored this aspect in HPyVs. Relative synonymous codon usage (RSCU) is an indicator of codon usage bias (CUB) and measures the ratio of each synonymous codon for a given amino acid (Xu et al., 2008). It has been proven that RSCU is correlated with gene expression (Yu et al., 2015; Liu, 2020), and the similarity between RSCU of genes of infectious agents and the genes of their host is a tool to explore the changes in the expression of genes in the host (Jitobaom et al., 2020; Nambou and Anakpa, 2020; Maldonado et al., 2021; Nambou et al., 2022). An adaptive translational correlation between human viruses and infected organs has been reported and suggested the involvement of the CUB in the control of host defense machinery (Alonso and Diambra, 2020; Jitobaom et al., 2020). Recently, RSCU similarity studies among the coronaviruses, influenza A viruses, and various other viruses have allowed the discovery of key genes tendentially driving the pathogenesis of these infections, and drug prediction based on these genes had been achieved (Chen et al., 2020; Nambou and Anakpa, 2020; Maldonado et al., 2021; Nambou et al., 2022). In this context, disclosing the analogies among the RSCU of viral genes and human genes may enlighten the pathogenesis of HPyVs. However, studies on the CUB of HPyVs are very scarce. It was previously reported that the CpG dinucleotides of HPyVs are depleted and that translational selection is the main factor shaping the CUB of these viruses (Upadhyay and Vivekanandan, 2015). The CUB of the large T antigen (LT-Ag) of HPyVs has been also reported (Cho et al., 2019). However, the CUB of other HPyV proteins has not been singularly reported. In addition, the similarities between the CUB of HPyV genes and that of the human genes has not been reported, which needs an in-depth investigation.

Therefore, in the present study, we aimed at investigating the similarities between the RSCU of HPyV genes and that of the human genome in order to uncover the translational interactome of HPyVs and explore their role in PVAN.

## Materials and methods

### Data acquisition

The whole human genome was from GENCODE (https://www.gencodegenes.org/human/). The coding sequences of human HPyV proteins were retrieved from the NCBI virus database (https://www.ncbi.nlm.nih.gov/labs/virus/vssi/) and included the sequences of different types of HPyVs (MCPyV, JCPyV, BKPyV, HPyV9, and TSPyV). These subtypes of HPyVs were found relevant for studying PVAN because BKPyV and JCPyV are associated with nephropathy, and MCPyV has been detected in urine and kidney; in addition, HPyV9 and TSPyV have been detected in the kidneys and viremia has been detected in renal transplant patients (Babel et al., 2011; Kantarci et al., 2011; van der Meijden et al., 2014; Yang et al., 2017; Caller et al., 2019; Hirsch and Randhawa, 2019; Zanella et al., 2021).

The gene expression datasets were obtained from the GEO database (https://www.ncbi.nlm.nih.gov/geo/) and included the data with accession numbers GSE72925 and GSE75693. The expression data from control samples and HPyV infected samples were used for the subsequent analyses.

### Relative synonymous codon usage analysis

The RSCU values of different codons of the HPyV CDS sequences and human CDS sequences was computed by running the R library “vhcub” (Anwar et al., 2019). RSCU represents the ratio of the frequency of a codon to be chosen among the synonymous codons encoding for a given amino acid to the expected frequency of this codon (Cho et al., 2019; Nambou and Anakpa, 2020; Nambou et al., 2022). A codon with an RSCU value higher than 1 indicates a positive CUB of this codon, whereas RSCU value lower than 1 indicate a negative CUB (Cho et al., 2019; Nambou and Anakpa, 2020; Nambou et al., 2022). The codons with no CUB have RSCU value of 1 and the codons with RSCU lower than 0.6 or greater than 1.6 indicate codon usage “underrepresentation” or codon usage “overrepresentation” (Tripp et al., 2013; Upadhyay and Vivekanandan, 2015; Starrett et al., 2019).

### Identification of human genes with CUB similar to HPyV CUB

The similarity of the RSCU of HPyV sequences and that of the human CDS sequences was analyzed by computing the Pearson correlation between these sequences based on the Hmisc package in R. The correlation of human genes with HPyV genes was deemed credible when the correlation coefficient r was > 0.5 at a p-value threshold of < 0.05.

### Differential expression analysis

The expression datasets were used for differential expression analysis in R using the limma and edgeR packages. Genes with log2 of foldchange higher than 1.4 or lower that -1.4 with p-value lower than 0.05 were considered as those differentially expressed between the control and the HPyV infected proteins. The ggplot2 package was used for generating the volcano plots while the heatmaps were obtained by running the ComplexHeamap library in R. The ven diagrams were calculated using the online tool (https://bioinformatics.psb.ugent.be/webtools/Venn/).

### Protein–protein interaction network of human genes with CUB similar to that of HPyVs

The protein–protein interaction (PPI) network sets of genes were built using the Search Tool for the Retrieval of Interacting Genes (STRING) (https://string-db.org/). The high confidence was set to 0.7 while other parameters were kept as default. The built PPI networks were downloaded and further visualized and analyzed using the Cytoscape 3.4.0 software (Cytoscape Consortium, SanDiego, CA, USA). The keys were retrieved by performing the MCODE analysis at a max depth = 100, node score cut-off ≥ 0.2, degree cut-off ≥ 2, and K-core ≥ 2.

### Functional enrichment analysis

The TCGAbiolinks package in R was used for analyzing and visualizing the functions of the genes based on the TCGAanalyze_EAcomplete and TCGAvisualize_EAbarplot functions. The significantly enriched terms were obtained when the adjusted p-values where < 0.05.

## Results

### A- and U-ending codons are preferentially used in the coding sequences of human HPyVs

**Figure 1:**
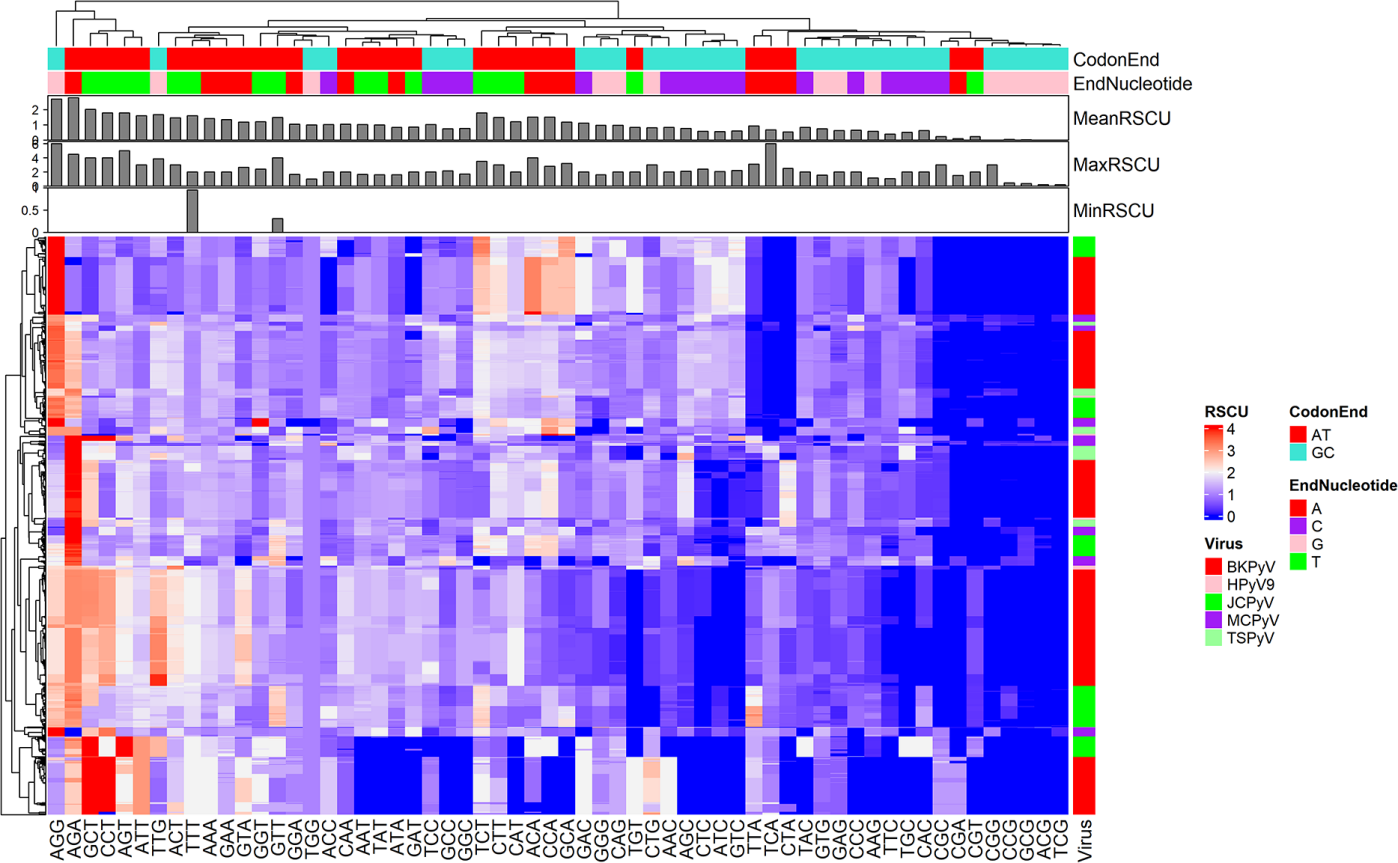
Heatmap showing the relative synonymous codons of human genes and genes of HPyVs.

To explore the CUB of the coding sequences of HPyVs, the RSCU values of codons in each CDS sequences of HPyVs was computed. The heatmap in **Figure 1** indicates the RSCU profile of the analyzed sequences. The A-/T-ending codons were the most preferentially used compared to the G-/C-ending codons in CDS sequences of HPyVs (**Figure 1**). The most preferentially used G-/C-ending codons were the AGG and TTG codons while the most preferentially used A-/T-ending codons were AGA, CCT, GCT, AGT, ATT, TCT, ACT, ATT, TTT, AAA, CTT, ACA, CCA, and GCA codons. The most underrepresented codons (RSCU values lower than 0.6) were the CGG, TCG, ACG, CCG, and GCG codons (**Figure 1**). The RSCU values of the codons of the CDS sequences of HPyVs varied between 0 and 6 (**Figure 1**). For some viral genes, differences were recorded among viruses while for other genes, strong similarities were recorded (**Figure 1**).

### Detection of human genes with CUB similar to the CUB of human HPyVs and functional enrichment analysis

**Figure 2:**
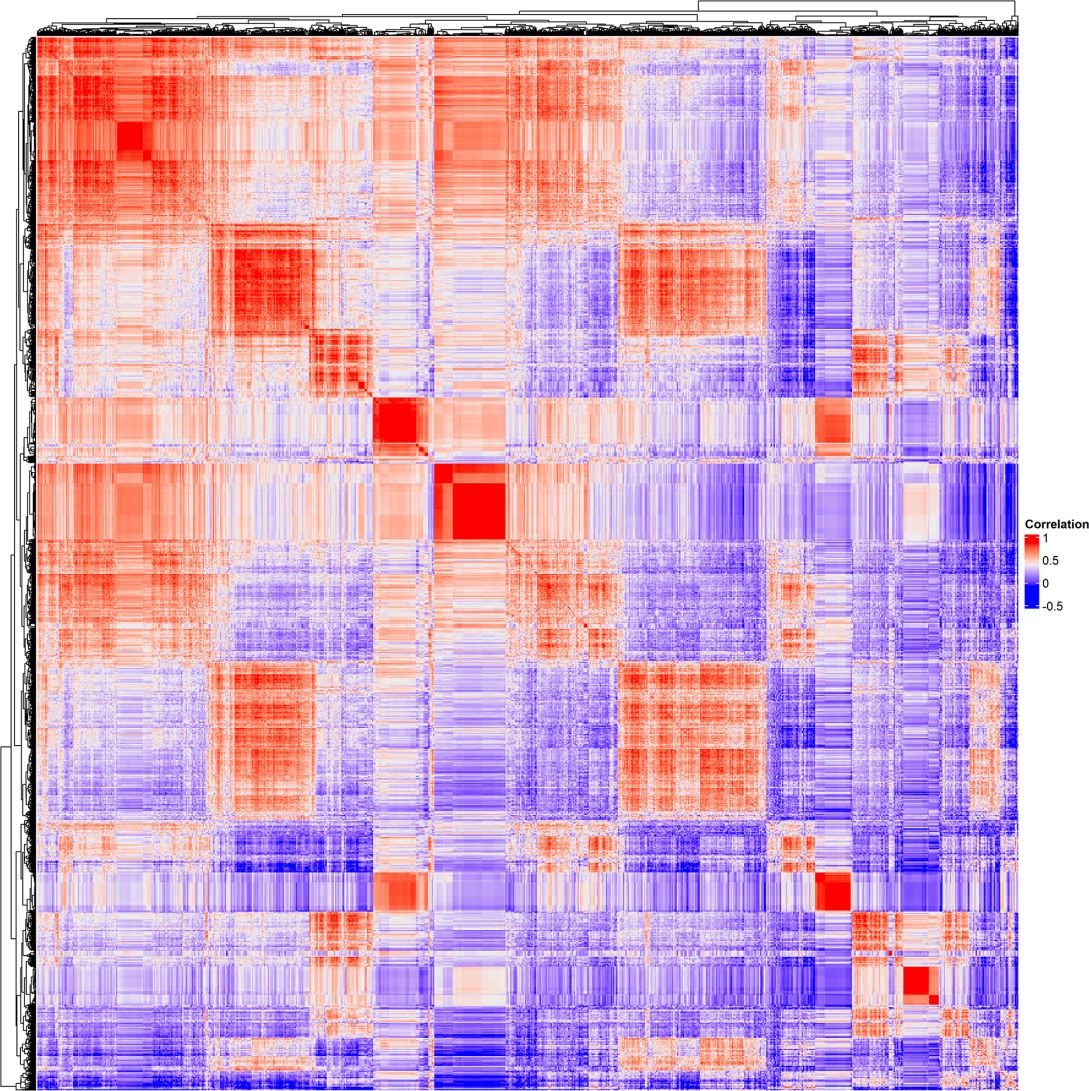
Correlation heatmap of genes of HPyVs and human genes with CUB similar to that of HPyVs.

**Figure 3:**
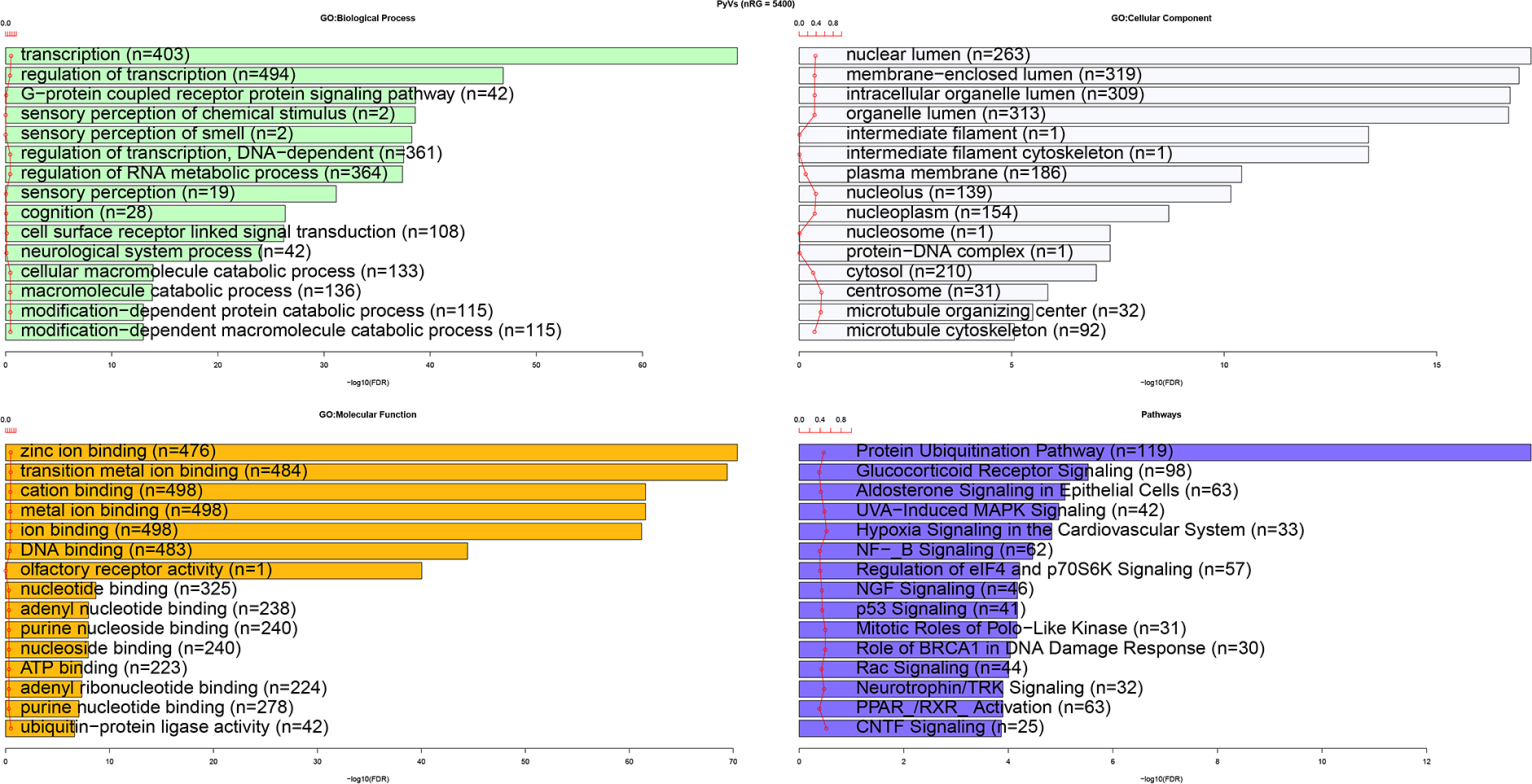
Functional enrichment analysis of human genes with CUB similar to that of HPyVs.

To discover human genes sharing similarities with CUB of genes of the HPyVs, the RSCU values of the codons of human genes were calculated and merged with those of genes of the HPyVs. Based on the screening criteria of p < 0.05 and r > 0.5 (**Supplementary File S1**), we identified a total of 5400 genes as those presenting highly significant similarities with the genes of HPyVs based on the RSCU values. The correlation heatmap of these similar genes was as depicted in **Figure 2**. To uncover the functions of the human genes with CUB similar to that of the HPyV genes, we performed GO (Gene Ontology) and KEGG (Kyoto Encyclopedia of Genes and Genomes) enrichment test. The results exhibited that the most prevalently enriched biological processes were transcription (403 genes) and regulation of transcription (494 genes), sensory perception of chemical stimulus (2 genes), G-protein coupled receptor protein signaling pathway (42 genes), sensory perception of smell (2 genes), sensory perception of smell (n=2)

sensory perception of chemical stimulus (n=2), DNA-dependent regulation of transcription (361 genes), and regulation of RNA metabolic process (364 genes) (**Figure 3**). For the category of molecular function, zinc ion binding (476 genes), transition metal ion binding (484 genes), cation binding (498 genes), metal ion binding (498 genes), ion binding (498 genes), and DNA binding (483 genes) were the most enriched terms whereas for cellular component, nuclear lumen (263 genes) and membrane-enclosed lumen (319 genes) were the most enriched terms (**Figure 3**). The most enriched pathways were protein ubiquitination pathway (119 genes), glucocorticoid receptor signaling (98 genes), and aldosterone signaling in epithelial cells (63 genes) (**Figure 3**).

### Protein–protein interaction network prediction and identification of hub clusters of the human genes with CUB similar to that of HPyVs and their functional roles

**Figure 4:**
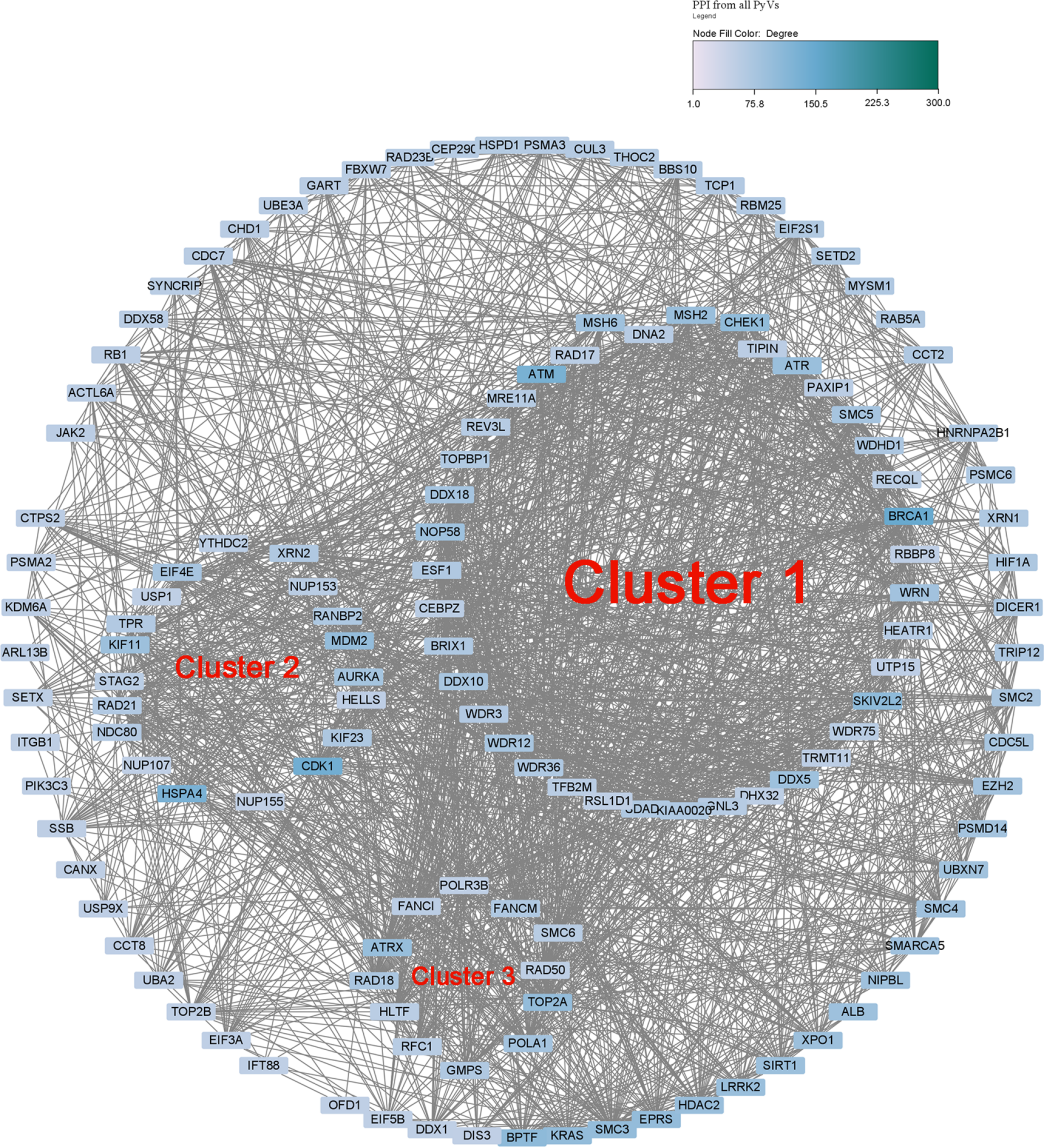
Protein-protein interaction network of human genes with CUB similar to that of HPyVs.

The PPI of human genes with CUB similar to that of the genes of HPyVs was constructed using the top-2000 genes based on the correlation rank (**Supplementary Figure S1**). The network showing nodes with degree greater or equal to 50 was as depicted in **Figure 4**. The network diameter of the HPyV PPI network was 8 while its radius was 5; the number of connected components was 2, with 1938 nodes, 17796 edges, and the average number of neighbors of 18.383 (**Figure 4**). The nodes with degree higher or equal to 100 were BRCA1, CDK1, ATM, HSPA4, BPTF, SKIV2L2, CHEK1, and KRAS. Using the MCODE plugin, we identified 34 clusters. Cluster 1 with the highest score (26.038) was composed of 53 proteins as key hub genes, with BRCA1, ATM, SKIV2L2, CHEK1, MSH2, ATR, NOP58 and DDX5 as the most important genes (**Figure 4**). The functional analysis indicated that these key hub genes were participating in the biological processes of response to DNA damage stimulus (10 genes), cellular response to stress (12 genes), and RNA processing (10 genes) (**Supplementary Figure S2**). In the category of cellular component, membrane−enclosed lumen (16 genes), organelle lumen (16 genes), intracellular organelle lumen (16 genes), nuclear lumen (17 genes), and nucleolus (15 genes) were the most enriched while the most predominant molecular function terms were helicase activity (seven genes) and ATP−dependent helicase activity (six genes) (**Supplementary Figure S2**). The pathways associated with the key hub genes were role of BRCA1 in DNA damage response (eight genes), role of CHK proteins in cell cycle checkpoint control (six genes), hereditary breast cancer signaling (seven genes), and p53 signaling (six genes) (**Supplementary Figure S2**). The second cluster (Cluster 2, score of 13.633) contained 50 proteins, with CDK1, HSPA4, MDM2, KIF11, AURKA, EIF4E, KIF23, RANBP2 and RAD21 as the most important hub proteins. The genes in cluster 2 were involved in the biological processes of mRNA transport (10 genes), RNA localization (10 genes), and nucleic acid transport (10 genes) (**Supplementary Figure S3**). The cellular component terms associated with these genes were pore complex (nine genes), nuclear pore (nine genes), nuclear envelope (nine genes), and non−membrane−bounded organelle (16 genes) while the most prevalent molecular functions were nucleocytoplasmic transporter activity (three genes), RNA cap binding (two genes), microtubule motor activity (three genes) and damaged DNA binding (two genes) (**Supplementary Figure S3**). The pathways associated with these genes were those involved in mitotic roles of Polo−Like kinase (six genes), protein ubiquitination pathway (five genes), cleavage and polyadenylation of pre−mRNA (two genes), cell cycle: G2/M DNA damage checkpoint regulation (two genes), aldosterone signaling in epithelial cells (three genes), and ATM signaling (two genes) (**Supplementary Figure S3**). The third cluster (Cluster 3) contained 23 proteins among which 12 had node degrees higher than 50; TOP2A, ATRX and POLA1 were the nodes with the highest degrees. The biological processes related to the 23 proteins in cluster 3 were cellular response to stress (12 genes), DNA repair (nine genes), response to DNA damage stimulus (nine genes), DNA metabolic process (nine genes), and DNA replication (four genes); terms such as positive regulation of viral genome replication (one gene) and immune system development (two genes) were also recorded (**Supplementary Figure S4**). Replication fork (three genes) was the most enriched cellular component while nucleotidyltransferase activity (three genes) was the most enriched molecular function (**Supplementary Figure S4**). The most enriched pathways were DNA double−strand break repair by homologous recombination (four genes), DNA double−strand break repair by non−homologous end joining (four genes), hereditary breast cancer signaling (six genes), role of BRCA1 in DNA damage response (five genes), DNA double−strand break repair by homologous recombination (three genes), and role of CHK proteins in cell cycle checkpoint control (three genes) (**Supplementary Figure S4**).

### Involvement of human genes with CUB similar to that of HPyV genes in PVAN

**Figure 5:**
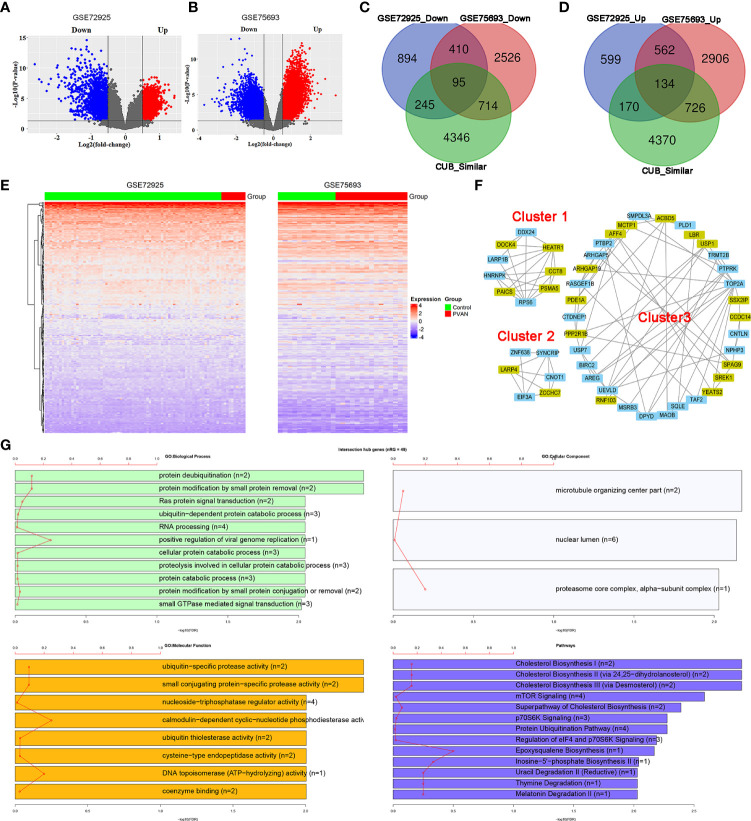
Involvement of human genes with CUB similar to that of human HPyVs in PVAN. **(A)** Volcano plot showing differential expression analysis of genes in GSE72925. **(B)** Volcano plot showing differential expression analysis of genes in GSE75693. **(C)** Venn diagram showing the intersection between downregulated DEGs from GSE72925, downregulated DEGs from GSE75693 and human genes with CUB similar to that of HPyV genes. **(D)** Venn diagram showing the intersection between upregulated DEGs from GSE72925, upregulated DEGs from GSE75693 and human genes with CUB similar to that of HPyV genes. **(E)** Heatmaps of the expression profile of the 229 human genes with CUB similar to that of HPyV genes that were consistently and differentially expressed in GSE72925 and GSE75693. **(F)** Hub genes of the PPI network of human genes with CUB similar to that of HPyV genes that were consistently and differentially expressed in GSE72925 and GSE75693 from the first three cluster generated by MCODE. The green nodes indicate downregulated DEGs while the other nodes were upregulated DEGs. **(G)** Functional enrichment of hub genes of the PPI network of human genes with CUB similar to that of HPyV genes that were consistently and differentially expressed in GSE72925 and GSE75693.

In order to explore the involvement of human genes with CUB similar to that of HPyV genes in PVAN, the GSE72925 and GSE75693 datasets were retrieved and the differential expression analysis was performed between the uninfected normal samples and PVAN kidney tissue. From the GSE72925 dataset, as indicated in **Figure 5A** and **Supplementary File S2**, we detected 3109 differentially expressed genes (DEGs) with 1465 of them being upregulated while the other 1644 genes were downregulated. From the GSE75693 dataset, as indicated in **Figure 5B** and **Supplementary File S3**, we detected 8073 DEGs with 4328 of them being upregulated while the other 3745 genes were downregulated. Further, we intersected the list of upregulated and downregulated DEGs from both GSE72925 and GSE75693 datasets with the list of human genes with CUB similar to that of HPyV genes. As shown in **Figure 5C**, 95 DEGs were found as the intersection between downregulated DEGs from both GSE72925 and GSE75693 and human genes with CUB similar to that of HPyV genes. Moreover, as shown in **Figure 5D**, 134 DEGs were found as the intersection between upregulated DEGs from both GSE72925 and GSE75693 and human genes with CUB similar to that of HPyV genes. Thus, a total of 229 DEGs were identified as key genes which can be considered as credible human genes whose translation can be affected by the coadaptation of human with HPyVs. The heatmaps showing the expression of these genes in GSE72925 and GSE75693 were depicted in **Figure 5E**. These 229 intersection key DEGs were involved in the biological processes of transcription (28 genes), regulation of RNA metabolic process (26 genes) DNA−dependent regulation of transcription (25 genes), regulation of transcription (30 genes), glycerophospholipid metabolic process (five genes), and glycerolipid metabolic process (five genes); the biological processes related to virus (regulation of defense response to virus by virus (two genes), and regulation of defense response to virus (two genes)) were also enriched (**Supplementary Figure S5**). Nuclear chromosome (five genes) was the most enriched cellular component while zinc ion binding (36 genes) and transition metal ion binding (36 genes) were the most enriched molecular functions (**Supplementary Figure S5**). The most enriched pathways were Cholesterol Biosynthesis I (two genes), Cholesterol Biosynthesis II (*via* 24,25−dihydrolanosterol) (two genes), and Cholesterol Biosynthesis III (*via* Desmosterol) (two genes) (**Supplementary Figure S5**).

The PPI network of the 229 proteins was constructed and indicated strong interactions among these proteins (**Supplementary Figure S6**). This PPI network contained 219 nodes and 1091 edges; the average number of neighbors was 9.963 while the network diameter and radius were 7 and 4, respectively (**Supplementary Figure S6** **)**. The MCODE plugin allowed the retrieval of 8 hub proteins (PAICS, DDX24, HNRNPK, HEATR1, ARP1B, RPS6, CCT8, DOCK4, and PSMA5) from cluster 1, 6 hub proteins from cluster 2 (CNOT1, SYNCRIP, LARP4, EIF3A, and ZCCHC7) and 34 hub proteins from cluster 3 (**Figure 5F**). Functional enrichment analysis indicated that these 49 hub proteins were involved in the biological processes of protein deubiquitination (two genes), protein modification by small protein removal (two genes), Ras protein signal transduction (two genes), ubiquitin−dependent protein catabolic process (three genes), RNA processing (four genes), and positive regulation of viral genome replication (one genes) (**Figure 5G**). The most enriched cellular components were microtubule organizing center part (two genes), nuclear lumen (six genes) and alpha−subunit complex of proteasome core complex (one gene) while the most enriched molecular functions were ubiquitin−specific protease activity (two genes), small conjugating protein−specific protease activity (two genes), and nucleoside−triphosphatase regulator activity (four genes) (**Figure 5G**). The pathways associated with the hub genes were predominantly cholesterol biosynthesis (two genes), cholesterol biosynthesis II (*via* 24,25−dihydrolanosterol) (two genes), cholesterol biosynthesis III (*via* Desmosterol) (two genes), mTOR Signaling (four genes), superpathway of cholesterol biosynthesis (two genes), p70S6K signaling (three genes), protein ubiquitination pathway (four genes), regulation of eIF4 and p70S6K signaling (three genes) (**Figure 5G**).

## Discussion

In the present study, we aimed to identify human genes with CUB similar to that of HPyV genes and explore their potential involvement in the pathogenesis of PVAN. The RSCU analysis indicated that the A- and T-ending codons are more preferentially used in HPyV genes. In addition, significant positive correlations in CUB among HPyV genes and human genes were recorded. In total, 5400 human genes were correlated to the HPyV genes. Gene expression analysis indicated that 229 of these genes were consistently and differentially expressed between normal kidney tissues and kidney tissues from patients with nephropathy. The PPI network indicated strong interactions between these proteins. Functional enrichment analysis indicated that these genes were involved in biological processes of transcription, and regulation of transcription and pathways related to protein ubiquitination pathway, inflammation and immune system. The identified genes may serve as diagnostic biomarkers and potential therapeutic targets for PVAN.

In the present study, we analyzed the RSCU of the viral genes from five HPyVs. The study indicated that the A- and T-ending codons were those preferentially used by the HPyVs. This result indicated that the preferred codons were influenced by compositional constraints (Mazumder et al., 2018). Moreover, these results suggested that the CUB of HPyVs is shaped by the occurrence of selection constraints, as it was previously reported (He et al., 2016; Majeed et al., 2020).

The interference of host cells with viruses is pathological or harmful for the host, inducing cell morphological changes and dysregulation of gene expression in the host cell (Lyles, 2000). It has been reported that, following viral infection, host gene expression is co-transcriptionally or post-transcriptionally downregulated by the viruses, which is essential for their own replication (Herbert and Nag, 2016; McFadden et al., 2021). Studies indicate that the mechanism underlying this regulatory effect is the consequence of the interaction of viral proteins with cellular protein (Maginnis, 2018). The host ribosomes are used by both the virus and host cells for translation of host or viral mRNAs (Herbert and Nag, 2016). The protein synthesis in the host is affected by its response to virus and the translation of viral mRNAs is influenced by the co-adaptation of the virus with the host due to the use of the same translational apparatus (Herbert and Nag, 2016). The CUB of hosts and viruses is indicative of the evolutionary variations, including adaption, evasion from the host’s immune system and survival (Butt et al., 2016; Kumar et al., 2018; Maginnis, 2018). However, the effect of CUB on gene transcription following the infection by HPyVs has not been reported so far. In the present work, we found that the list of human genes with codon usage similar to that of HPyVs were implicated in transcription and regulation of transcription. Thus, the present findings implied that the HPyVs hijack the transcriptional regulation in human cells, thus leading to pathogenetic changes. Studies indicate that transcription plays a significant role in the initiation of viral infection (Lyles, 2000). Since transcription-related processes were those most significantly enriched, we anticipated that the disturbance of transcriptional processes is significantly important in HPyV infections and might be a crucial initiation step in the pathogenesis. Several studies have shown that the HPyVs are oncogenic (Prado et al., 2018), however, their molecular mechanism is unelucidated. In the present study, we found that human genes with CUB similar to that of HPyVs were involved in various cancer pathways. This support the hypothesis that the HPyVs are oncogenic. In addition, pathways of immune and inflammatory responses were enriched, showing that HPyVs may activate inflammatory responses and weaken immune system in human, thus allowing the occurrence of pathogenetic processes. In addition, the cell apoptosis related pathways were also enriched. These functional changes enlightened our knowledge on the molecular mechanism of HPyVs.

The molecular mechanism of PVAN is not well elucidated. Up to date, only few studies have explored the molecular profile changes in the kidney tissues of patients with PVAN. Single-cell transcriptomics of the cellular response to BKPyV has been performed recently (An et al., 2021). Proteomic studies also indicated that HPyV infection triggers G2 arrest of the cell cycle and evasion of the virus to innate immune system (Caller et al., 2019). TaqMan low density array was also used to analyze the expression profiles of 90 immune-related genes in kidney graft biopsy; the results disclosed various biological networks-related to BKPyV infection (Girmanova et al., 2012). HPyV T Antigen has been also reported to regulate the APOBEC3B (Starrett et al., 2019). Pathological and bioinformatics analyses indicated that genes regulated by BK HPyV infection are involved in cytoplasmic vacuolation implicating endoplasmic reticulum stress with DDIT3 as the key hub gene (Zhao et al., 2022). The analysis of BK PVAN transcriptome was unable to uncover BK PVAN-specific molecular signatures, which limits the clinical application of the corresponding findings (Pan et al., 2018). Though molecular and transcriptomic studies have helped discovering the gene expression changes and potential molecular mechanisms, much is left to be discovered regarding the molecular mechanism of PVAN. In the present study, we found that the co-adaptation of HPyVs with human may impact the gene replication and expression in human due to the competitive use of codons present in the cellular environment. This approach has been used in previous research studies which have been demonstrated useful in disclosing interesting molecular aspects involved in infectious diseases such as human infections of coronaviruses and influenza viruses (Nambou and Anakpa, 2020; Nambou et al., 2022). Here, we found that 5400 human genes had CUB similar to that of HPyVs. These genes can be considered as the translational interactome of HPyV genes. The implication of these genes in PVAN was further confirmed by differentially expression analysis indicating the differential expression of 229 human genes with CUB similar to that of HPyV genes; these genes were consistently downregulated or upregulated across two datasets. These results indicated that the evolutionary coadaptation of human and HPyVs instigates differential gene expression and functional dysregulation in human, which leads to the pathogenetic occurrence of PVAN. Our study indicated that the 229 DEGs with CUB similar to that of HPyVs constituted a strong PPI network and the eight hub genes of this network were enriched in the biological processes related to transcription, regulation of RNA metabolic process, metabolism-related processes, regulation of defense response to virus and response to biotic stimulus. This suggested that coadaptation of HPyVs with human is a key component involved in the pathogenic mechanism of the infection by HPyVs in PVAN. Our results support the recent works showing that coadaptation of coronaviruses and influenza viruses induces abnormal gene expression in human (Nambou and Anakpa, 2020; Nambou et al., 2022). It was also similar to another work showing that human genes with CUB similar to coronaviruses could impact protein expression in human (Maldonado et al., 2021). Thus, the identified genes have the potential to serve as diagnostic biomarkers and potential therapeutic targets for PVAN, which needs additional characterization. In addition, we found that a large portion of human genes with CUB similar to that of HPyVs were differentially expressed among the PVAN and the control uninfected samples, confirming their involvement in PVAN. Nonetheless, other genes were not identified as differentially expressed genes, and may constitute new findings revealed in this study. Further experimental characterization of these genes is necessary to confirm their role in PVAN.

In conclusion, this study explored the molecular mechanism of HPyV infections based on the CUB similarities and confirmed their involvement in PVAN. These genes may serve as diagnostic biomarkers and therapeutic targets or clues for further mechanism research, but experimental validation is required.

## Data availability statement

Publicly available datasets were analyzed in this study. This data can be found here: The human genome used in this study can be found at GENCODE (https://www.gencodegenes.org/human/). The coding sequences of human polyomavirus proteins can be downloaded from the NCBI virus database (https://www.ncbi.nlm.nih.gov/labs/virus/vssi/). The gene expression datasets GSE72925 and GSE75693 are available in the GEO database (https://www.ncbi.nlm.nih.gov/geo/). Some of the data analysis results files are available in this manuscript as supplementary files, the other huge files can be provided by the corresponding author upon reasonable requirement.

## Author contributions

YF: Conceptualization, Methodology, Formal analysis, Investigation, Writing – original draft, Funding acquisition. DG: Investigation, Writing – review and editing. SZ: Investigation, Writing – review and editing, Funding acquisition. QW: Investigation, Writing – review and editing, Funding acquisition. YL: Supervision, Writing – review and editing. TL: Supervision, Writing – review and editing, Funding acquisition. All authors contributed to the article and approved the submitted version.

## Funding

This work was supported by the Natural Science Foundation of China [grant number 81870513]; A Special Supportive Program for Organ Transplantation by COTDF [grant number 2019JYJH08]; National Clinical Research Center for Geriatrics, West China Hospital, Sichuan University [grant number Z20201011] and the West China Nursing Discipline Development Special Fund Project, Sichuan University [grant number HXHL21014].

## Acknowledgments

We are thankful to the Natural Science Foundation of China, A Special Supportive Program for Organ Transplantation by COTDF, National Clinical Research Center for Geriatrics, West China Hospital, Sichuan University, and the West China Nursing Discipline Development Special Fund Project, Sichuan University which supported this study through their funds.

## Conflict of interest

The authors declare that the research was conducted in the absence of any commercial or financial relationships that could be construed as a potential conflict of interest.

## Publisher’s note

All claims expressed in this article are solely those of the authors and do not necessarily represent those of their affiliated organizations, or those of the publisher, the editors and the reviewers. Any product that may be evaluated in this article, or claim that may be made by its manufacturer, is not guaranteed or endorsed by the publisher.

## Supplementary material

Supplementary Figure 1 PPI of human genes with CUB similar to that of the genes of HPyVs was constructed using the top-2000 genes based on the correlation rank.

Supplementary Figure 2 Functional enrichment analysis of human genes with CUB similar to that of HPyVs in cluster 1.

Supplementary Figure 3 Functional enrichment analysis of human genes with CUB similar to that of HPyVs in cluster 2.

Supplementary Figure 4 Functional enrichment analysis of human genes with CUB similar to that of HPyVs in cluster 3.

Supplementary Figure 5 Functional enrichment analysis of human genes with CUB similar to that of HPyVs overlapped with differential expression genes in GSE72925.

Supplementary Figure 6 Functional enrichment analysis of human genes with CUB similar to that of HPyVs overlapped with differential expression genes in GSE75693.

Supplementary File S1 List of human genes with CUB similar to that of HPyVs based on the Pearson correlation analysis.

Supplementary File S2 List of DEGs from GSE72925.

Supplementary File S3 List of DEGs from GSE75693.
