# Supplementary figures and images for: Human genes with relative synonymous codon usage analogous to that of polyomaviruses are involved in the mechanism of polyomavirus nephropathy
Source: Front Cell Infect Microbiol. 2022 Sep 8;12:992201. doi: 10.3389/fcimb.2022.992201 (PMC9492876; doi:10.3389/fcimb.2022.992201)

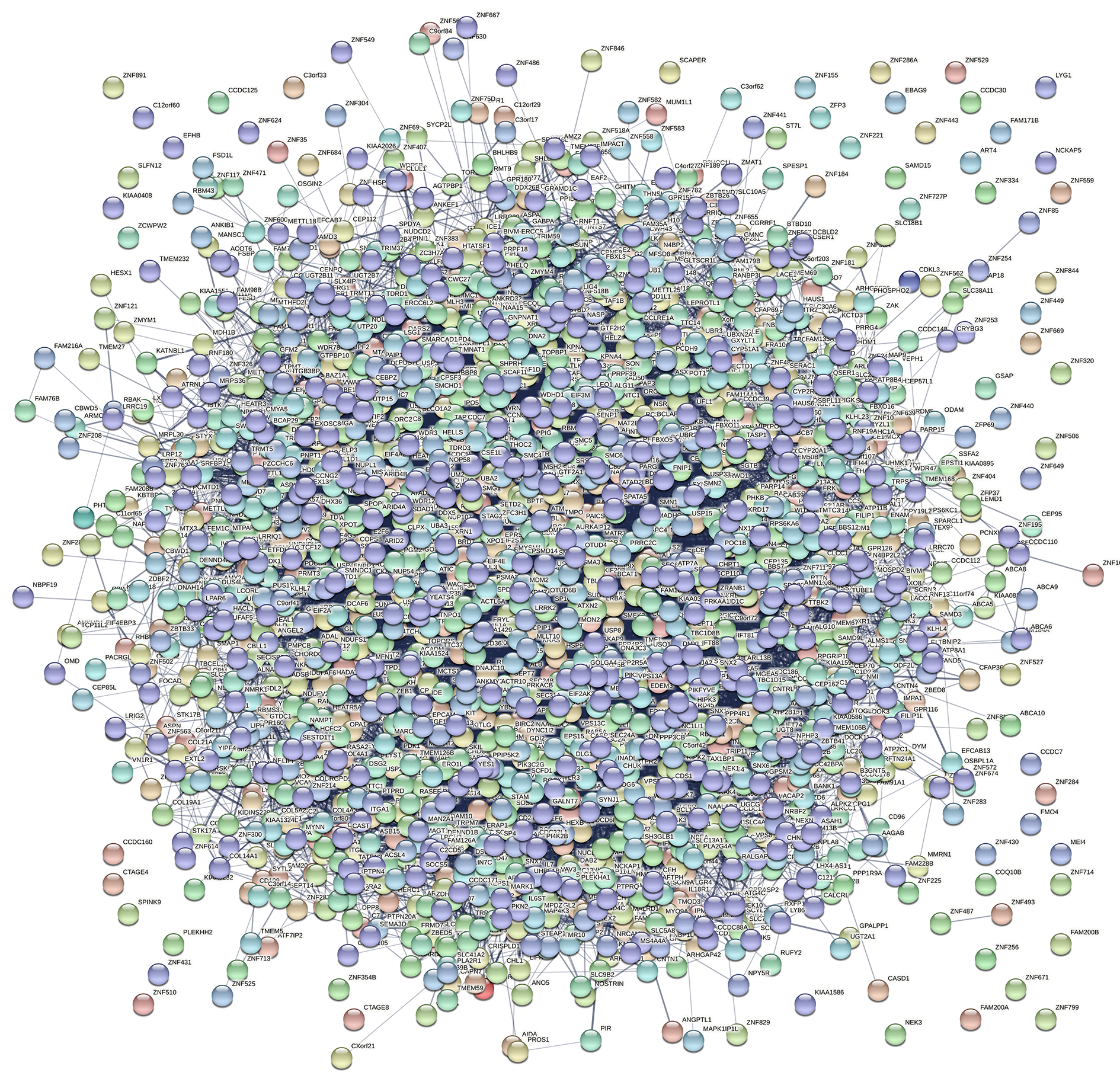

Supplement: Supplementary Figure 1 — PPI of human genes with CUB similar to that of the genes of HPyVs was constructed using the top-2000 genes based on the correlation rank. [file Image_1.tif]

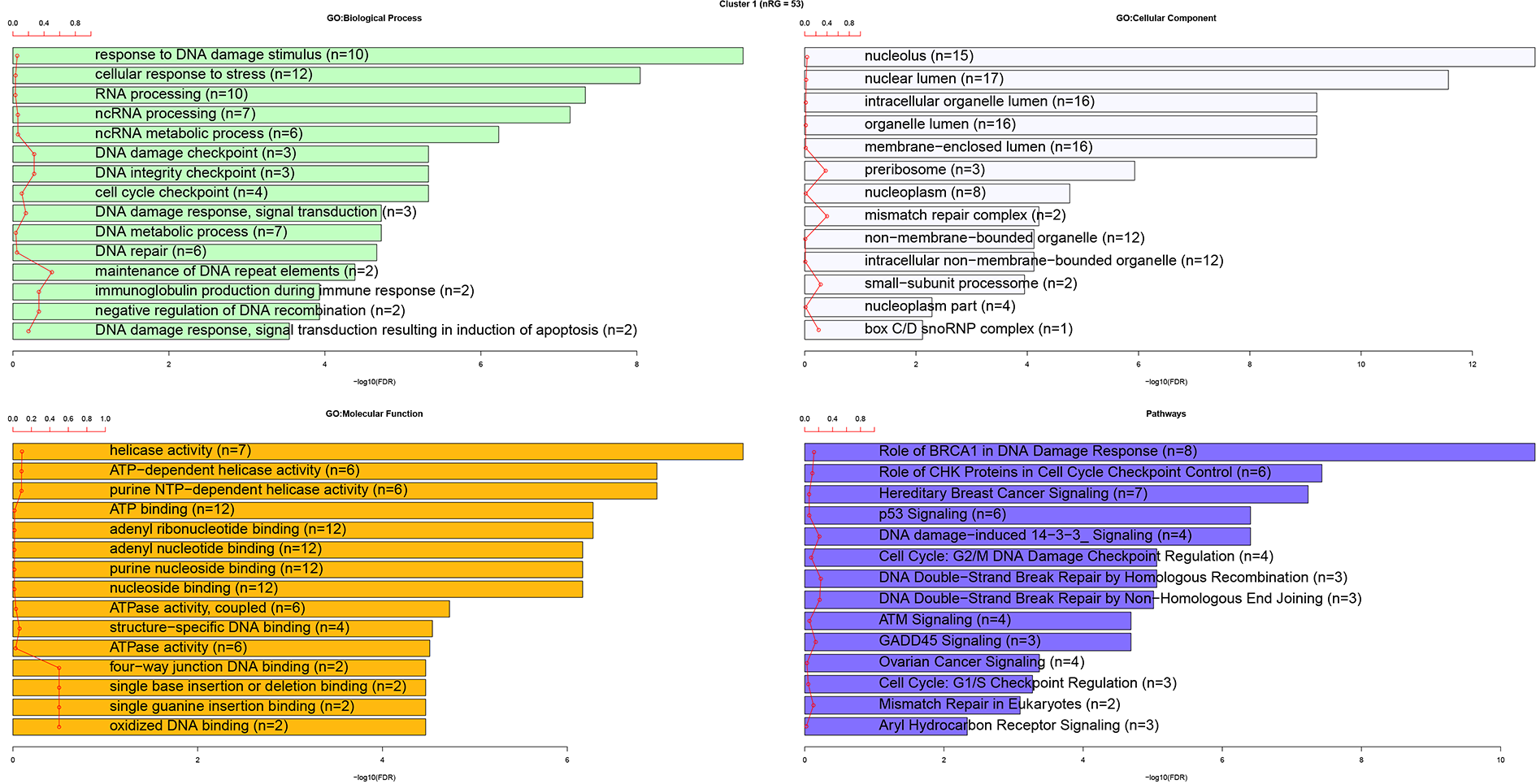

Supplement: Supplementary Figure 2 — Functional enrichment analysis of human genes with CUB similar to that of HPyVs in cluster 1. [file Image_2.tif]

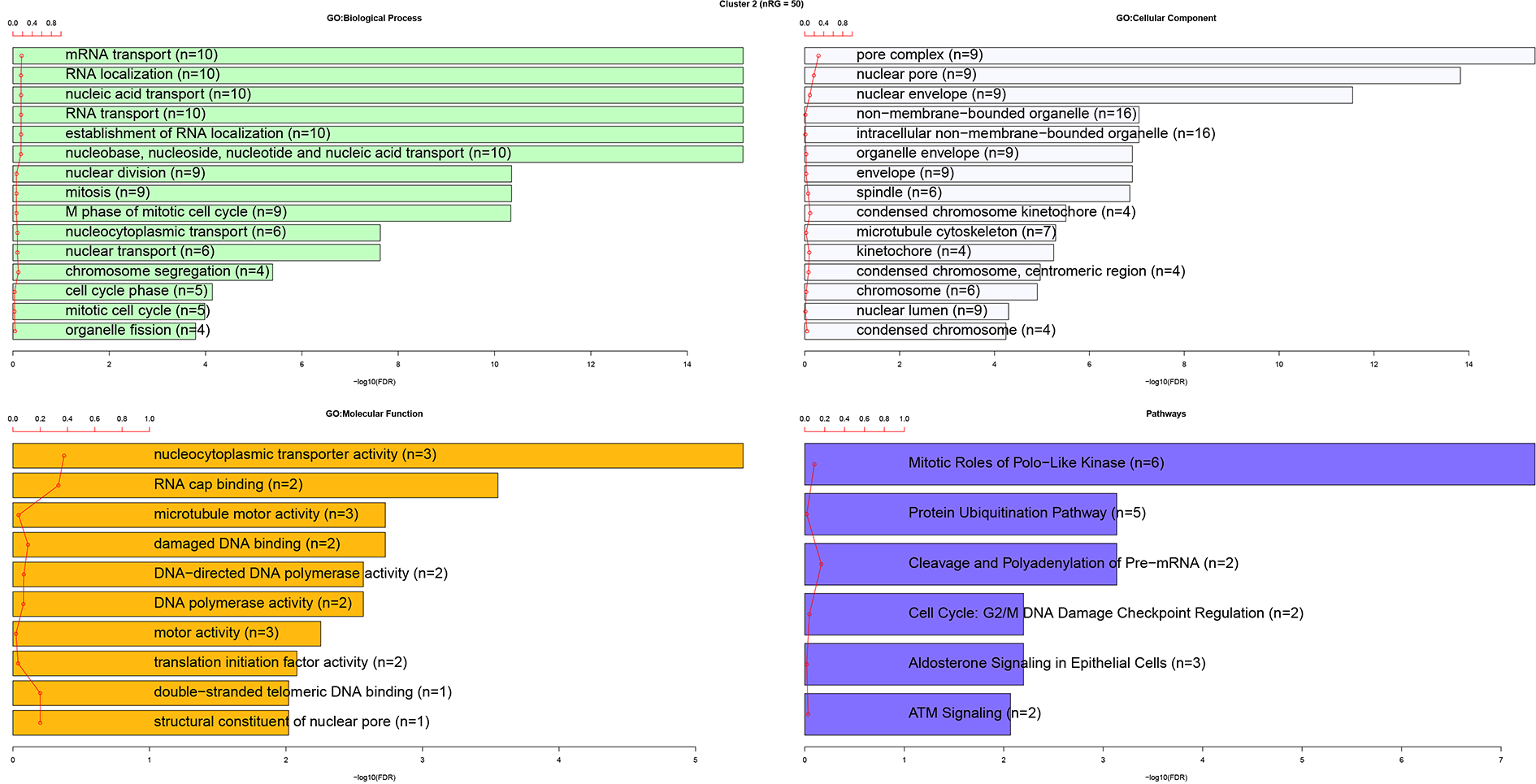

Supplement: Supplementary Figure 3 — Functional enrichment analysis of human genes with CUB similar to that of HPyVs in cluster 2. [file Image_3.tif]

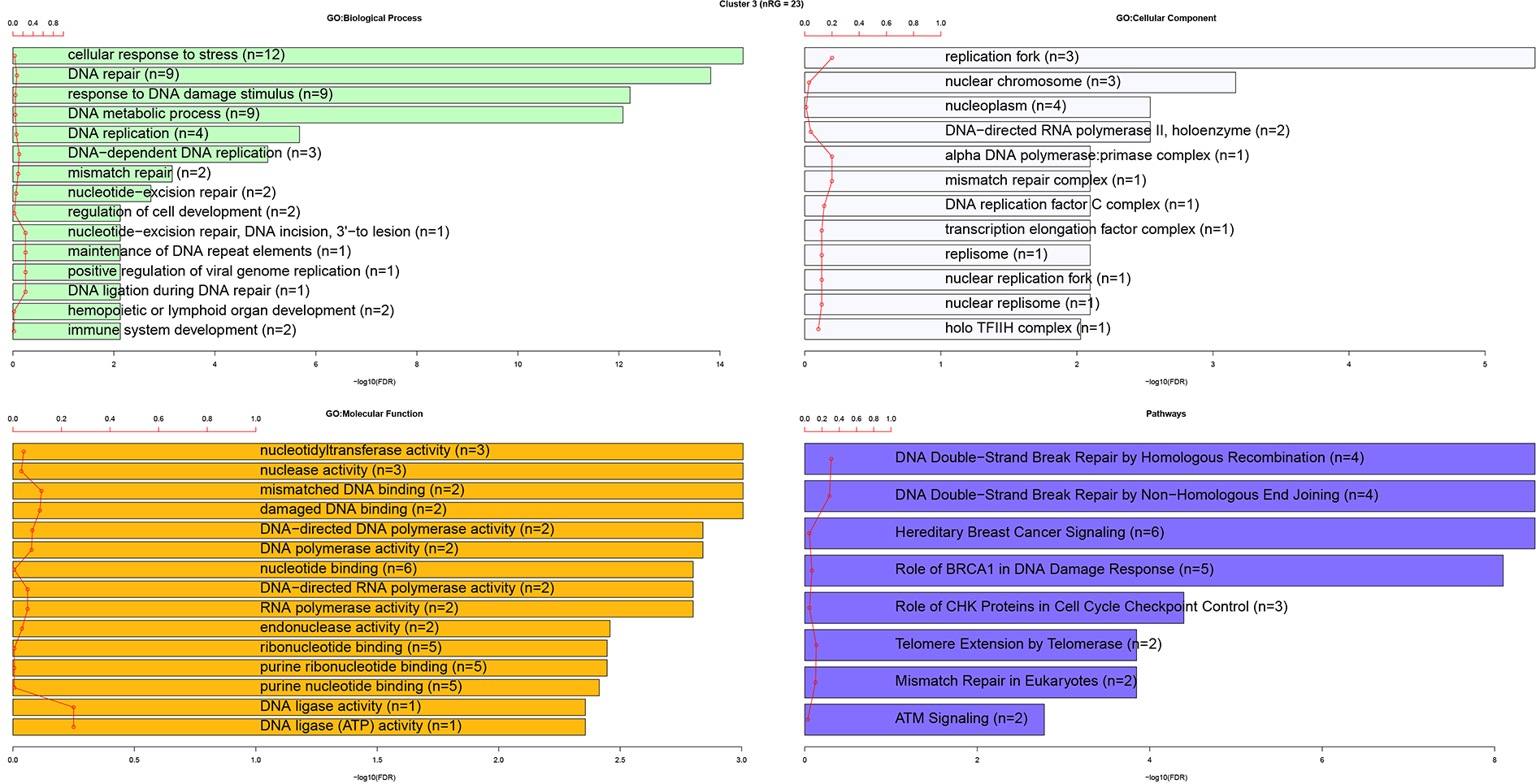

Supplement: Supplementary Figure 4 — Functional enrichment analysis of human genes with CUB similar to that of HPyVs in cluster 3. [file Image_4.tif]

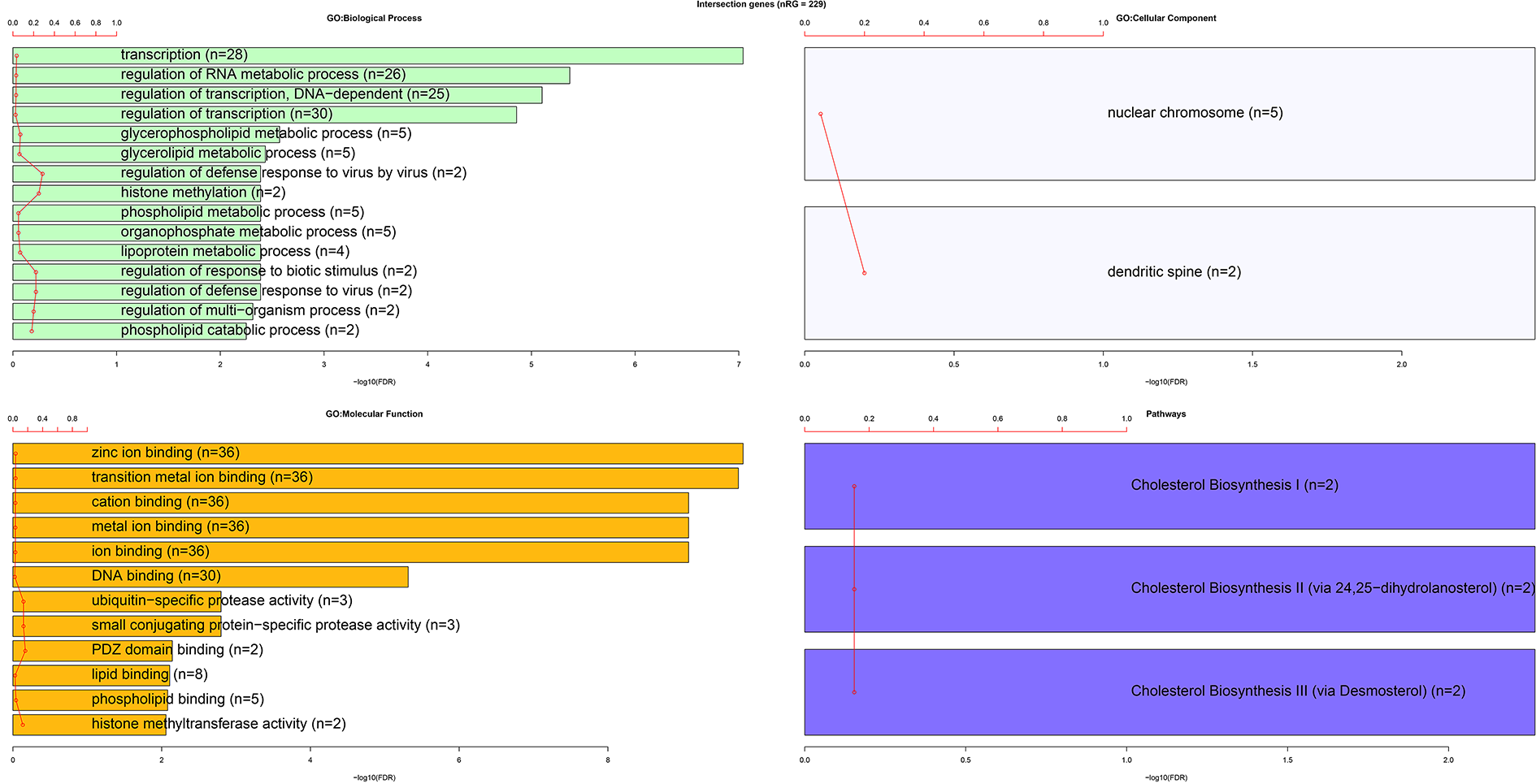

Supplement: Supplementary Figure 5 — Functional enrichment analysis of human genes with CUB similar to that of HPyVs overlapped with differential expression genes in GSE72925. [file Image_5.tif]

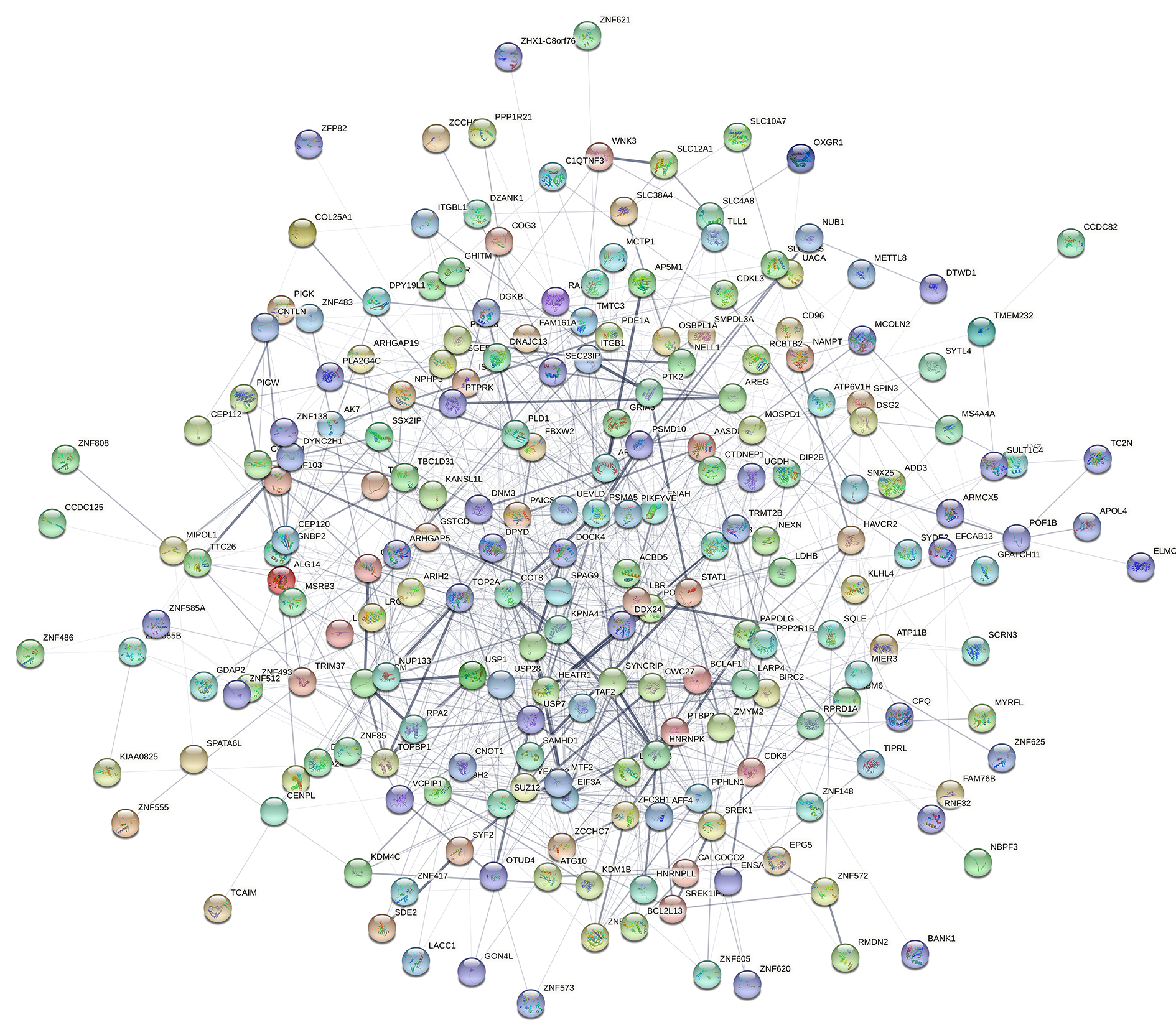

Supplement: Supplementary Figure 6 — Functional enrichment analysis of human genes with CUB similar to that of HPyVs overlapped with differential expression genes in GSE75693. [file Image_6.tif]
